# Pervasive influence of maternal and paternal criminal offending on early childhood development: a population data linkage study

**DOI:** 10.1017/S0033291716003007

**Published:** 2016-11-29

**Authors:** K. R. Laurens, S. Tzoumakis, M. Kariuki, M. J. Green, M. Hamde, F. Harris, V. J. Carr, K. Dean

**Affiliations:** 1 School of Psychiatry, University of New South Wales, Sydney, Australia; 2 Schizophrenia Research Institute, Sydney, Australia; 3 Neuroscience Research Australia, Sydney, Australia; 4 Department of Psychiatry, School of Clinical Sciences, Monash University, Melbourne, Australia; 5 Justice Health & Forensic Mental Health Network, Matraville, NSW, Australia

**Keywords:** Cognition, physical health, psychopathology, social–emotional function, violent offending

## Abstract

**Background:**

Parental criminal offending is an established risk factor for offending among offspring, but little evidence is available indicating the impact of offending on early childhood functioning. We used data from a large Australian population cohort to determine associations between exposure to parental offending and a range of developmental outcomes at age 5 years.

**Method:**

Multi-generation data in 66 477 children and their parents from the New South Wales Child Development Study were combined using data linkage. Logistic and multinomial regressions tested associations between any and violent offending histories of parents (fathers, mothers, or both parents) obtained from official records, and multiple measures of early childhood developmental functioning (social, emotional–behavioural, cognitive, communication and physical domains) obtained from the teacher-reported 2009 Australian Early Development Census.

**Results:**

Parental offending conferred significantly increased risk of vulnerability on all domains, particularly the cognitive domain. Greater risk magnitudes were observed for offending by both parents and by mothers than by fathers, and for violent than for any offending. For all parental offending exposures, vulnerability on multiple domains (where medium to large effects were observed) was more likely than on a single domain (small to medium effects). Relationships remained significant and of comparable magnitude following adjustment for sociodemographic covariates.

**Conclusions:**

The effect of parental offending on early childhood developmental outcomes is pervasive, with the strongest effects on functioning apparent when both parents engage in violent offending. Supporting affected families in early childhood might mitigate both early developmental vulnerability and the propensity for later delinquency among these offspring.

## Introduction

Multi-generation data from studies using American, Australian, British, Dutch and Swedish samples, and a variety of methodologies, consistently indicate increased risk for juvenile delinquency and adult criminal offending among the offspring of parents who engage in criminal behaviour (Farrington *et al.* 2001; van de Rakt *et al.* 2008; Bijleveld & Wijkman, 2009; Farrington *et al.* 2009; Nijhof *et al.* 2009; Frisell *et al.* 2011; Goodwin & Davis, 2011; Beaver, 2013; Kendler *et al.* 2015), with greatest risk associated with violent parental offending (Kendler *et al.* 2015). These offspring represent a high-risk population that might be targeted with interventions to interrupt the intergenerational transmission of criminal behaviour; preventative efforts delivered prior to the offspring's engagement in crime are likely to yield greater success than later interventions (Junger *et al.* 2013). Considering that the peak age of onset for offending is 8–14 years (Moffitt, 1993; Farrington, 2003), and the prevalence of offending is greatest during adolescence (Loeber *et al.* 2012), early childhood (0–5 years) represents a prime window for delivery of preventative intervention. Family/parent training programmes provided during this period can effectively avert antisocial behaviour and delinquency (Piquero *et al.* 2016), but identifying a broader range of early childhood targets for preventative intervention might further mitigate these outcomes for the offspring of offending parents.

Most multi-generation studies investigating the impact of parental offending have focused specifically on offspring offending outcomes during adolescence and adulthood, but in younger samples, there is evidence of intergenerational transmission of antisocial behaviour more broadly. Multiple studies have demonstrated the association between parental antisocial behaviour and childhood conduct/externalizing problems among their offspring (Rhule *et al.* 2004; Smith & Farrington, 2004; Thornberry *et al.* 2009; Raudino *et al.* 2013), including as early as 18 months of age (Kim *et al.* 2009). These externalizing problems are important early risk markers of later offending, with cross-national data demonstrating that persisting childhood conduct problems among boys, particularly physical aggression, increase the risk of adolescent violent and non-violent delinquency (Broidy *et al.* 2003). The effect of parental antisocial behaviour on offspring is not limited to externalizing difficulties, however, with several studies also demonstrating effects on internalizing (i.e. anxiety and depression) alongside externalizing problems (Herndon & Iacono, 2005; Coley *et al.* 2011).

Further, parental offending may have an impact on offspring cognitive functioning. A study using linked register data in a Swedish sample comprising more than a million male military conscripts and their fathers indicated an association between fathers’ criminal-conviction status and their sons’ cognitive ability at age 18 years (Latvala *et al.* 2015), though no association was identified between parental offending and academic performance in a smaller sample of a thousand American youth aged between 7 and 16 years (Murray *et al.* 2012). For the offspring of incarcerated parents, adverse outcomes have been described spanning multiple domains of developmental function, including behavioural, emotional, social, cognitive and physical outcomes (Seymour, 1998), but it is unknown whether such pervasive effects might also be observed for parental offending more generally (i.e. including non-custodial sentences) and without the additional effects of parental separation entailed in custodial sentencing. The importance of assessing functioning across this broader range of developmental domains is underscored by longitudinal prospective cohort investigations (e.g. Niarchou *et al.* 2015; Poulton *et al.* 2015), which demonstrate that early childhood difficulties in behavioural, emotional, social, cognitive and physical functioning are risk factors for a variety of adverse adolescent and adult outcomes beyond juvenile delinquency and adult offending, including mental and physical illnesses, social maladjustment, and poor educational and occupational outcomes.

Given the dominant influences of parents, especially mothers, in their offspring's lives during early childhood, parental criminal offending might have a particularly pervasive impact on developmental outcomes during this period. Most previous investigations have considered only the impact of fathers’ offending on offspring behaviour, as the lower prevalence of crime among women limits power to determine associations between maternal offending and offspring outcomes, and most ‘high-risk’ samples have been convened on offending males only. However, the importance of considering maternal offending is underscored by emerging evidence that the impact of mothers’ and fathers’ antisocial behaviour on child behaviour may be mediated through different pathways (Thornberry *et al.* 2009), and that sons and daughters may be differentially affected by their parents’ behaviour (Kim *et al.* 2009; Auty *et al.* 2017). Interestingly, a recent study that examined the effect of engagement in crime by both parents, as well as paternal and maternal offending separately, indicated comparable levels of risk for criminal offending among offspring of two criminal parents as those conferred by a criminal father or mother alone (Beaver, 2013). Nonetheless, the risks conferred by antisocial mothers and fathers for antisocial behaviour in their offspring are independent (i.e. these risks remain significant after controlling for the co-parents’ antisociality) (Blazei *et al.* 2006).

The present study uses data from a large Australian epidemiological sample of 66 477 children (Carr *et al.* 2016) to investigate the association of parental offending (by fathers, mothers and both parents) with a range of early childhood developmental indices, namely, social, emotional–behavioural, cognitive, communication and physical functions measured at the age of 5 years. We sought particularly to determine the pervasiveness of vulnerabilities across the range of developmental domains among offspring. The effects of both history of any form of offending by parents and history of violent offending specifically were examined separately, and analyses were adjusted for various sociodemographic covariates that might influence associations between parental offending and child developmental outcomes. We hypothesized that, relative to children with no parental history of criminal offending, children with a parental history of any offending would show increased risk of dysfunction across the variety of developmental domains. We further anticipated that children of offending parents would be at greater risk of demonstrating vulnerability on multiple developmental domains than any single domain. We also hypothesized that greater risk magnitudes would be associated with maternal than with paternal offending, and for violent offending than any offending.

## Method

### Study population and data linkage

Data were drawn from the New South Wales Child Development Study (NSW-CDS; http://nsw-cds.com.au/; Carr *et al.* 2016), which uses multi-agency data linkage to combine population records for a cohort of 87 026 children and their parents. Linkage of data from early childhood (birth to 5 years) was completed in 2014 by an independent agency, the Centre for Health Record Linkage (http://www.cherel.org.au/), using probabilistic linkage methods and with adherence to strict privacy protocols (researchers received de-identified records only). Matching variables included name, date of birth, residential address and sex (for detail on linkage methods and data collections, see Carr *et al.* 2016). Ethical review was conducted by the NSW Population and Health Services Research Ethics Committee (HREC/11/CIPHS/14), with data custodian approvals granted by the relevant government departments.

The NSW-CDS cohort encompasses 99.7% of children who entered full-time schooling (kindergarten) in NSW in 2009, at around 5 years of age. For these children, teachers at all government and private schools who had a minimum of 1 month's knowledge of the child completed the Australian Early Development Census (AEDC; https://www.aedc.gov.au/; Brinkman *et al*. 2012, 2014). Via linkage of the child's AEDC record with data from the NSW Register of Births, Deaths, and Marriages – Birth Registrations for the period spanning 1 January 2000–31 December 2006, parental records were obtained for 72 245 children (83.0% of the cohort) whose births were registered in NSW. Among these, AEDC information on developmental functioning was unavailable for 3129 children with special needs (i.e. a chronic medical, physical, or intellectually disabling condition requiring special assistance in the classroom).

### Measures

#### Early childhood development outcomes

The AEDC is a population assessment of early childhood functioning, with established reliability and validity for measurement of five domains of development (Janus *et al.* 2011; Brinkman *et al.* 2014), including: social competence (SOCIAL), emotional maturity (EMOTIONAL), language and cognitive skills (COGNITIVE), communication skills and general knowledge (COMMUNICATION), and physical health and wellbeing (PHYSICAL). A detailed description of the developmental competencies measured by each domain is provided in Table 1. Two sets of outcome variables were derived for analysis in the present study: (i) for each of the five AEDC developmental domains, a dichotomous variable distinguished developmentally ‘vulnerable’ children (those scoring within the bottom 10% of the national AEDC population distribution) from the remaining 90% of children classified as ‘not vulnerable’; and (ii) a variable summarizing the number of AEDC domains on which children scored in the vulnerable range (range 0–5).

**Table 1.** Description of early childhood developmental domain outcomes, as measured by the teacher-reported 2009 AEDC

AEDC domain	Domain description
Social competence (SOCIAL)	Overall social competence, responsibility and respect, approach to learning (e.g. works independently and adapts to routines), and readiness to explore new things (e.g. books, toys, games)
Emotional maturity (EMOTIONAL)	Pro-social and helping behaviours, anxious and fearful behaviour, aggressive behaviour, and hyperactivity and inattention
Language and cognitive skills (COGNITIVE)	Basic literacy, advanced literacy, basic numeracy, and interest in literacy, numeracy and memory
Communication skills and general knowledge (COMMUNICATION)	Broad developmental competencies and skills in communication and general knowledge (e.g. understands and uses language effectively)
Physical health and wellbeing (PHYSICAL)	Gross and fine motor skills, physical independence, and physical readiness for the school day (e.g. tired, hungry, or unkempt)

AEDC, Australian Early Development Census.

#### Parental offending exposures

Data for these variables were obtained from NSW Bureau of Crime Statistics and Research (http://www.bocsar.nsw.gov.au/) records, which includes information on court appearances for charges before the Local, District, Supreme and Children's Criminal Courts for the period spanning 1 January 1994–31 December 2009^1^†. These records capture adult, but not adolescent, offending for most parents in our cohort [mean ages of fathers and mothers at January 1994 were 23.0 (s.d. = 6.4) and 20.1 (s.d. = 5.5) years, respectively]. We derived two types of offending exposure variables: (i) a dichotomous variable coding parental history of any offence, relative to children with no history of any parental offending; and (ii) a dichotomous variable coding parental history of violent offending^2^, also relative to no parental offending. The ‘any’ and ‘violent’ offending exposures were derived based on paternal, maternal and biparental offending (both parents) to generate a total of six parental offending variables for analysis. Data from children (*n* = 2639; 3.8%) with a parental history of attendance at court under non-criminal regulations only (e.g. dispute of fines and penalties such as those issued for traffic violations) rather than criminal acts were excluded from statistical analyses. Thus, the final cohort for this project comprised 66 477 children (online Supplementary Fig. S1).

#### Sociodemographic covariates

Demographic variables considered *a priori* as potential confounders or mediators of the associations between parental offending history and child developmental outcomes included: (i) child sex, (ii) child age at time of AEDC assessment (three levels: <5, 5, and >5 years), (iii) maternal age at child's birth (three levels: <26, 26–36, and >36 years), (iv) English spoken as a second language (excluding children fully proficient in English, and coded dichotomously: no/yes), and (v) socio-economic status (SES; coded dichotomously: disadvantaged/not disadvantaged). SES was based on the Socio-Economic Indexes for Areas (SEIFA; Pink, 2013) measure of the average income and employment status for each residential postcode in Australia. SEIFA quintiles were recoded from the AEDC national quintile scores, and dichotomized into disadvantaged (quintiles 1 and 2) and not disadvantaged (quintiles 3, 4 and 5). All sociodemographic variables were obtained from the AEDC data collection, with the exception of maternal age at child's birth (obtained from the NSW Register of Births, Deaths, and Marriages – Birth Registrations and the NSW Ministry of Health Perinatal Data Collection, for the period spanning 1 January 2000–31 December 2006)^3^.

### Statistical analysis

Data analysis was conducted using IBM SPSS version 23.0 (IBM, 2015). A series of bivariate (unadjusted) and multivariable (adjusted for covariates) logistic regression analyses examined the pattern and magnitudes of association between the six exposure variables (any and violent offending by fathers, mothers and both parents) and the five AEDC developmental outcomes. These analyses provided odds ratios (ORs) and their 95% confidence intervals (CIs) as measures of effect size, with ORs of 1.00 to 2.00 (or 1.00 to 0.50) interpreted as small in magnitude, 2.00 to 5.00 (or 0.50 to 0.20) interpreted as medium, and >5.00 (or <0.20) as large (Rosenthal, 1996). Results were statistically significant if the 95% CI did not cross 1.00.

A series of multinomial regression analyses investigated the strength of the associations between the six exposure variables (any and violent offending by fathers, mothers and both parents) and the number of AEDC domains on which children presented vulnerability (six levels for any offending: 0–5 domains; four levels for violent offending, due to reduced numbers: 0–3 or more domains). Unadjusted associations, and associations following adjustment for covariates, were examined.

#### Supplementary analyses^4^

A series of supplementary analyses were conducted to confirm the presence of associations when exposure or outcome variables were entered into statistical models simultaneously (i.e. to identify independent effects after accounting for any shared variance between exposures and between outcomes). The major analyses were also repeated separately for girls and boys to confirm that any associations observed were present in both sexes^5^.

### Ethical standards

All procedures contributing to this work comply with the ethical standards of the relevant national and institutional committees on human experimentation and with the Helsinki Declaration of 1975, as revised in 2008.

## Results

### Sample characteristics

The prevalence of the parental offending exposures, child developmental vulnerability outcomes and sociodemographic covariates for the sample are provided in Table 2. Histories of any and violent offending by fathers were three and four times more prevalent, respectively, than those by mothers. There were 46 669 children (67.5%) with no parental offending history (these constituted the reference group for all analyses). The numbers of children presenting vulnerability on each of the five AEDC domains ranged from 5.4% (COGNITIVE) to 8.3% (SOCIAL), with the prevalence of each domain within 1.0% of the rate in the full NSW-CDS cohort (Carr *et al.* 2016). Vulnerability on a single AEDC domain (10.5%) only marginally exceeded the number of children vulnerable on multiple domains (9.6%).

**Table 2.** Prevalence of parental history of any and violent offending (exposure variables), early childhood developmental vulnerability (outcome variables), and sociodemographic covariates in the sample of 66 477 children and their parents

Study variables	*n* (%)
Exposure variables: parental offending history	
Parental history of any offending	
Father	17 631 (26.5)
Mother	5775 (8.7)
Both parents	3598 (5.4)
Parental history of violent offending	
Father	6853 (10.3)
Mother	1724 (2.6)
Both parents	870 (1.3)
No parental history of offending	46 669 (70.2)
Outcome variables: developmental vulnerability^a^	
Vulnerability on AEDC developmental domains	
Social competence	5549 (8.3)
Emotional maturity	4734 (7.1)
Language and cognitive skills	3589 (5.4)
Communication skills and general knowledge	5480 (8.2)
Physical health and wellbeing	5467 (8.2)
Number of AEDC domains of vulnerability	
1	6950 (10.5)
2	3309 (5.0)
3	1648 (2.5)
4	1013 (1.5)
5	451 (0.7)
Sociodemographic covariates	
Child sex, female	32 852 (49.4)
Child speaks English as a second language	10 319 (15.5)
Child age at time of AEDC assessment	
<5 years	2887 (4.3)
5 years	52 776 (79.4)
>5 years	10 814 (16.3)
Maternal age at child's birth^b^	
<26 years	14 727 (22.2)
26–36 years	44 442 (66.9)
>36 years	7308 (11.0)
Socio-economic status	
SEIFA categories 1 and 2: disadvantaged	30 122 (45.3)
SEIFA categories 3, 4 and 5: not disadvantaged	36 346 (54.7)

AEDC, Australian Early Development Census; SEIFA, Socio-Economic Indexes for Areas.

a Missing data on the individual AEDC domain outcome variables ranged between 169 and 411 (0.3–0.6%), and totalled 168 (0.3%) on the number of AEDC domains of vulnerability.

b Average maternal and paternal ages at child's birth were 29.8 (s.d. = 5.5) and 33.3 (s.d. = 6.4) years, respectively; for analyses adjusted for covariates, 542 (0.8%) missing data on this variable were assigned to the 26–36 years group.

### Association of parental offending and specific domains of functioning

#### Any offending

**Fig. 1.**
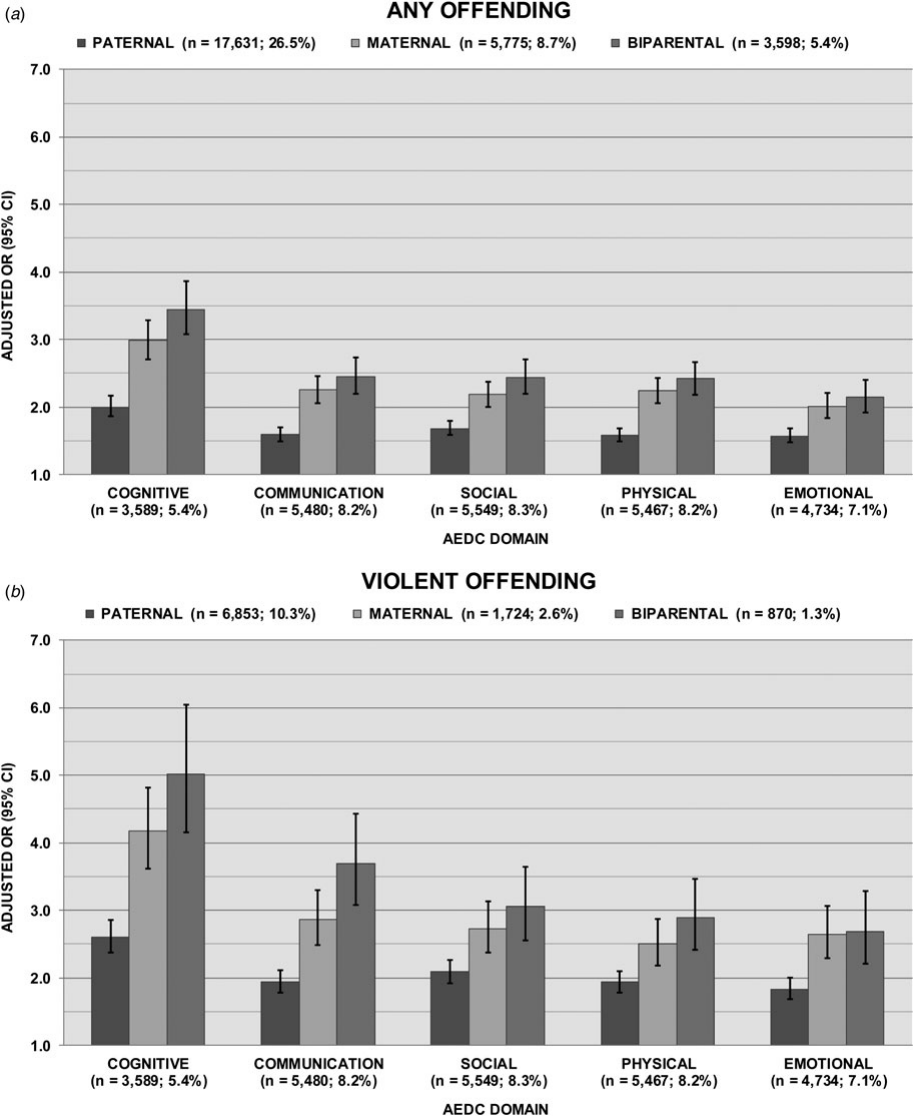
Associations between (*a*) any offending and (*b*) violent offending histories (paternal, maternal, biparental) and vulnerability on the five early childhood developmental domains. Values are odds ratios (ORs), with 95% confidence intervals (CIs) represented by vertical bars. AEDC, Australian Early Development Census.

Fig. 1*a* illustrates the significant associations of any offending by fathers, mothers and both parents with each of the five AEDC domains [the figure presents adjusted ORs (aORs) and their 95% CIs; full details on unadjusted ORs and aORs are provided in online Supplementary Table S1]. All associations remained significant following adjustment for sociodemographic covariates. For paternal offending, the magnitudes of the adjusted associations were small for each domain except COGNITIVE, where a medium magnitude of effect was observed (aOR range across domains: 1.57–2.00). Medium magnitudes of effect characterized the associations of maternal offending with all domains (aOR range: 2.01–2.99), and also for biparental offending (aOR range: 2.15–3.45). Thus, the strength of the associations observed between history of any parental offending and AEDC outcomes increased in the pattern: paternal < maternal < biparental offending. The lack of overlap between the CIs (Fig. 1*a*) suggests that the impact of maternal offending on early childhood development outcomes is significantly stronger than that of paternal offending, but that the magnitudes of effect associated with biparental offending are not significantly greater than those obtained for maternal offending (CIs overlap). For all three categories of parental offending, the smallest magnitude of effect was observed for the EMOTIONAL domain, and the greatest for the COGNITIVE domain.

#### Violent offending

The magnitudes of the associations between violent parental offending and the AEDC domains were greater than those observed for any offending, but they followed a similar pattern of increasing magnitude: paternal < maternal < biparental violent offending (Fig. 1*b*; and online Supplementary Table S1 for unadjusted ORs and aORs). The magnitudes of association for paternal violent offending ranged from small to medium (aOR range: 1.83–2.60), were all of medium magnitude for maternal violent offending (aOR range: 2.50–4.17), and ranged from medium to large for biparental violent offending (aOR range: 2.69–5.02). As for any offending, non-overlapping CIs for the paternal and maternal violent offending exposures implied significantly greater impact on development by maternal offending, but the risk of vulnerability associated with biparental violent offending was not significantly greater than that associated with maternal violent offending. Again, the COGNITIVE domain was characterized by the greatest magnitudes of association with violent offending by fathers, mothers and both parents. Similarly, the EMOTIONAL domain was characterized by the smallest magnitudes of association, with the exception that the association between maternal violent offending and the PHYSICAL domain was smaller.

#### Supplementary analyses

When considered separately, the significant associations between any and violent parental offending histories and child developmental vulnerabilities held both for girls and boys, and similarly increased in magnitude in the pattern paternal < maternal < biparental offending (online Supplementary Table S2).

As many children had both mothers and fathers with offending histories^6^, we examined whether independent associations remained for paternal and maternal offending when these exposures were entered concurrently into the statistical models (online Supplementary Table S3). Significant independent associations were observed between paternal and maternal offending (both any and violent) and each of the five AEDC domains. These effects were of small magnitude for both paternal and maternal offending, with the exception of medium effect sizes for the relationships between any and violent maternal offending and the COGNITIVE domain.

Similarly, as many children experienced vulnerability in multiple domains^7^, we examined whether the associations between parental offending and each AEDC domain remained significant when the other AEDC domains were entered simultaneously into statistical models. Independent associations were observed between parental offending and each AEDC domain after accounting for the associations of offending with the other domains (online Supplementary Table S4). These associations were all small in magnitude, excepting the medium effect magnitudes between any and violent maternal and biparental offending and the COGNITIVE domain.

### Association of parental offending with the number of developmental domains indicated as vulnerable

#### Any offending

**Fig. 2.**
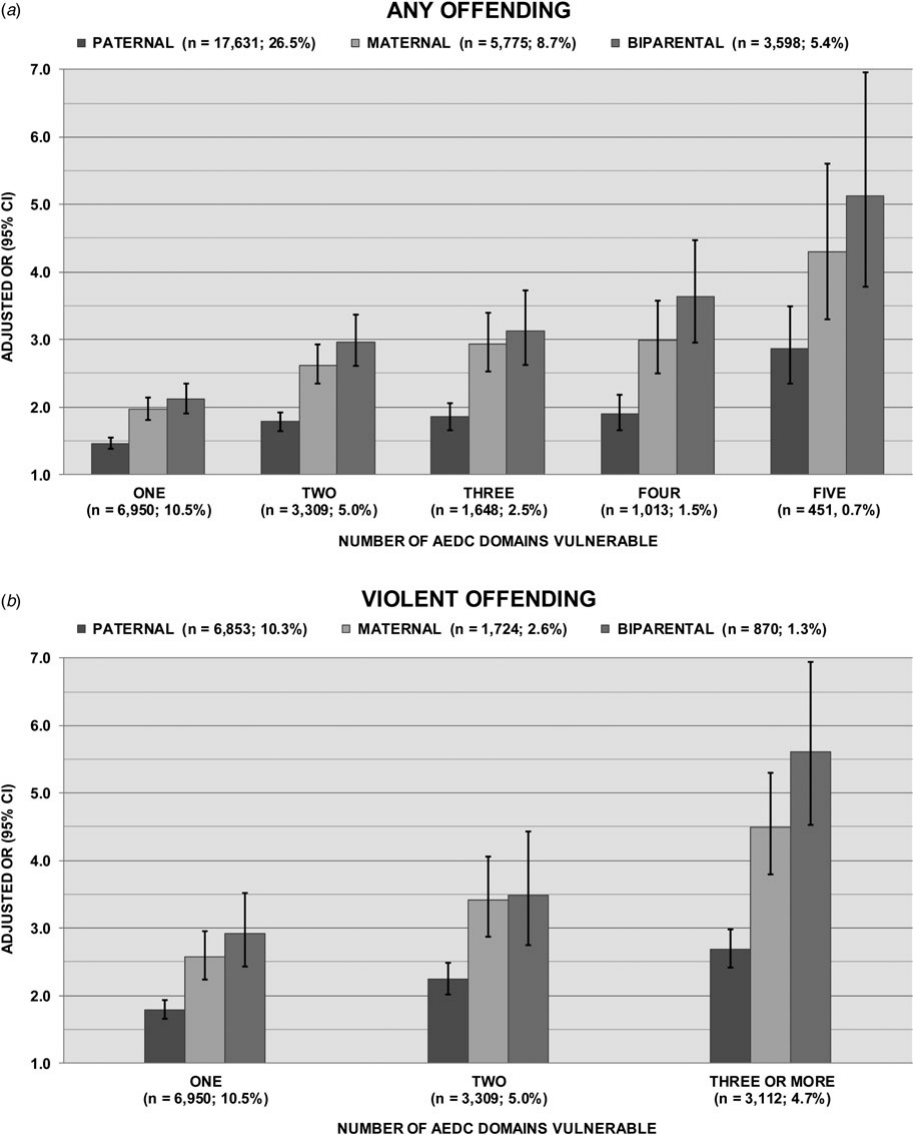
Associations between (*a*) any offending and (*b*) violent offending history (paternal, maternal, biparental) and the number of early childhood developmental domains on which children present vulnerability. Values are odds ratios (ORs), with 95% confidence intervals (CIs) represented by vertical bars. AEDC, Australian Early Development Census.

The associations between paternal, maternal and biparental history of any offending and the number of AEDC domains on which children presented vulnerability, adjusted for sociodemographic covariates, are illustrated in Fig. 2*a* (online Supplementary Table S5 details the unadjusted ORs and aORs). Adjustment for covariates effected little reduction in the associations. For all offending exposures, the magnitudes of association increased as the number of vulnerable domains increased. The magnitude of the associations also increased in the pattern paternal < maternal < biparental offending, with the greatest effect (large in magnitude; aOR = 5.45) observed between biparental offending and vulnerability on all five domains; however, non-overlapping CIs were observed only for paternal relative to maternal and to biparental offending (CIs for maternal and biparental offending overlapped).

#### Violent offending

Following adjustment for covariates, a similar pattern of increasing magnitudes of effect was observed between parental histories of violent offending and the number of AEDC domains of child vulnerability (Fig. 2*b*, and online Supplementary Table S5). Again, the associations were significant for paternal offending, larger in magnitude for maternal offending, and of greatest magnitude for biparental offending, with the largest effect (aOR = 5.61) apparent for the association between biparental history of violent offending and three or more domains of AEDC vulnerability; as previously, non-overlapping CIs were observed only for paternal relative to maternal and to biparental violent offending.

#### Supplementary analyses

Analyses conducted separately for female and male children (online Supplementary Table S6) indicated that these patterns of significant findings held both for girls and boys (due to reduced numbers, associations were computed for one, two, and three or more AEDC domains only).

## Discussion

In our NSW population sample of 66 477 children and their parents, offspring of offending parents were at greater odds of presenting vulnerability at age 5 years on measures of emotional–behavioural, social, cognitive, communication and physical functioning than children of non-offending parents, and, further, were more likely to present vulnerability on multiple domains than just a single domain. The magnitudes of these significant associations were greatest for violent parental offending, which aligns with investigations focused on offspring criminal behaviour (Kendler *et al.* 2015) and sons’ cognitive abilities (Latvala *et al.* 2015). For both the any and violent offending exposures, greater risk magnitudes were observed for offending by mothers and both parents (medium to large effects) than for offending by fathers (small to medium effects). The patterns held both for girls and boys.

Previous investigations have demonstrated the association of parental offending or antisocial behaviour on offspring behavioural (externalizing) and emotional (internalizing) problems during early and middle childhood (Herndon & Iacono, 2005; Coley *et al.* 2011). The small to medium magnitudes of associations observed between the parental offending exposures and the EMOTIONAL domain were comparable with or greater than those reported by others for externalizing and internalizing disorders at age 11 years (Herndon & Iacono, 2005), yet were the smallest among the five domains we assessed. The effects of parental offending on offspring were pervasive across emotional–behavioural, cognitive, social, communication and physical domains. The relative non-specificity of effects at age 5 years was also reflected in the greater likelihood for offspring of offenders to be affected on multiple domains than a single domain. This general impact of parental offending on early developmental functioning may reflect a pattern of vulnerability that is non-specific in early childhood, but may differentiate into particular patterns as the child grows older. Similarly, although the pervasive effect of parental offending across offspring developmental domains was observed for both girls and boys (with the associations for girls being consistently and significantly stronger than for boys only on the PHYSICAL domain), previous evidence of some sex-specific transmission of criminal convictions to daughters and sons via distinct mediating factors (Auty *et al.* 2017) also points to the potential emergence of greater specificity in the effects of parental offending as offspring get older. There may be distinct mechanisms contributing to the associations of offending with the various developmental domains, including different relative contributions of genetic and environmental effects, which may vary with age and sex (Blazei *et al.* 2006); these require an alternative (longitudinal twin) study design to disentangle.

In the context of this pervasive impact of parental offending on offspring early development, however, particularly pronounced effects were found on the COGNITIVE domain, which measured numeracy and literacy skills. Thus, the association demonstrated between fathers’ offending and their sons’ general cognitive ability at age 18 years (Latvala *et al.* 2015) is apparent already by school entry, and extends to maternal offending, and female as well as male offspring. This finding might reflect a particular capacity on the part of teachers to identify vulnerability in early literacy and numeracy skills relative to the other domains, or parental offending may particularly disadvantage development of these cognitive skills (e.g. through limited early exposure to written language and number concepts). The finding might also be explained by unmeasured parental cognitive abilities or genetic influences. Both genetic and environmental mechanisms contribute to parent–offspring transmission of criminal behaviour, with genetic effects constituting the more potent influence (Kendler *et al.* 2015). In the study by Latvala *et al.* (2015), genetic factors explained 80% of the association between fathers’ offending and sons’ cognitive ability; in our study, we cannot distinguish the relative contribution of genetic and environmental influences on the association between parental offending and the COGNITIVE (or other) domains. The association between academic underachievement in childhood (particularly in reading/literacy function) and antisocial behaviour problems is well established (Hinshaw, 1992) and appears to be reciprocally influenced and present during the first years of schooling (Trzesniewski *et al.* 2006). This implies a need to interrupt the association through preventative intervention delivered prior to school commencement, for example via pre-kindergarten programmes incorporating curricula and coaching in literacy, language and mathematics (Weiland & Yoshikawa, 2013).

Though the magnitude of effects reduced in supplementary analyses in which exposure or outcome variables were entered into statistical models simultaneously, the pattern of significant findings remained, confirming independent associations of maternal and paternal offending with vulnerability on every domain. Offending by both parents did not confer an increased risk relative to offending by mothers only, which has been observed elsewhere (Beaver, 2013); this may reflect the relatively low prevalence of biparental offending, limiting the precision of the effect estimation. Investigating the associations for maternal and biparental offending was facilitated by access to data from a large population sample of 66 477 children and their parents; such large samples are rare among investigations of offspring outcomes of parental offending (van de Rakt *et al.* 2008). Other strengths of the study relate to the use of linked administrative records from a population sample. Measurement of sensitive information such as history of criminal offending may be especially susceptible to sampling (selection and attrition) and information (recall and observer) biases, so data linkage of de-identified records provided a valuable means of accessing comprehensive data on both paternal and maternal offending at the population level while stringently protecting participants’ privacy and minimizing these biases. Use of official parental offending records and teacher-ratings of early childhood developmental functioning meant that exposure and outcome variables were collected independently of each other and neither was subject to parental perspective. Nonetheless, use of administrative records conferred several limitations. Access to offending records was restricted to 1994 onwards, thereby often excluding the peak age of offending and leading to likely underestimation of the magnitudes of effect of offending on early childhood development. In these official records, court appearances indexed offending, and omitted information about non-charged offences. This may be problematic if the likelihood of being detected and charged is not equal for all individuals; however, recent Australian data indicate moderate to substantial concordance between self-reported and officially recorded lifetime offending histories for most offence types (Payne & Piquero, 2016). The inclusion of information on various sociodemographic variables alleviated several sources of confounding, but other potential confounding variables were not available (e.g. individual-level SES data, parental education level, parental contact with child, and information on housing) or mediating variables (e.g. parenting styles). We had no method by which to confirm that children were in regular contact with their fathers and mothers; in 2012–2013, 75% of NSW children lived in intact families with both parents (Australian Bureau of Statistics, 2015). In terms of the potential cross-national generalizability of our findings, comparative published data on paternal and maternal offending rates are sparse. Rates of 18.0 and 7.2% were reported for fathers and mothers from official records on a Dutch town of 1681 families (Junger *et al.* 2013). Finally, the present study focuses on developmental outcomes assessed at one period only (early childhood); future linkages planned in this cohort will enable assessment of the persistence of these associations into middle childhood, adolescence and adulthood. In these future studies, we will differentiate proximal and distal impacts of parental offending occurring at particular periods in offspring's lives.

Identifying a broad range of early childhood developmental functioning associated with parental offending may offer new avenues for preventative interventions that could avert or mitigate a variety of adolescent and adult outcomes, including juvenile delinquency and adult offending. While there are already effective early childhood family/parent programmes that can prevent later antisocial behaviour and delinquency (Piquero *et al.* 2016), our study suggests that additional, child-centred targets for intervention may usefully augment these programmes. Pre-kindergarten programmes that support children's early development of literacy and numeracy skills (Weiland & Yoshikawa, 2013) might be particularly relevant. Intervention with children experiencing early childhood behavioural, emotional, social, cognitive and physical difficulties might also avert or mitigate later mental and physical illnesses, social maladjustment, and poor educational and occupational outcomes

## Notes

1 In the context of an unknown delay between commission of an offence and the associated court appearance, we elected to retain data from 188 children with a father, and 53 children with a mother, whose charge date occurred in the 5–7 months after AEDC collection (representing only 1.1 and 0.9% of children with offending fathers and mothers, respectively), as these offences were more likely than not to have occurred before AEDC assessment.

2 Violent offences represented a subset of offences incorporated within the any offending variable and included murder, attempted murder, manslaughter, assault resulting in serious injury, assault not resulting in serious injury, common assault, other acts intended to cause injury not elsewhere classified, abduction and kidnapping, deprivation of liberty/false imprisonment, aggravated sexual assault, non-aggravated sexual assault, non-assaultive sexual offences against a child, aggravated robbery, riot and affray.

3 Paternal age at child's birth (derived from the NSW Register of Births, Deaths, and Marriages – Birth Registrations) was not included in analyses due to multicollinearity with maternal age at birth (Pearson's *r* = 0.7).

4 Further analyses were undertaken after excluding children whose parents had received a custodial sentence, to remove the potential contribution of parental separation due to incarceration (excluding from analyses of any paternal, maternal and biparental offending, respectively: *n* = 1818 children of imprisoned fathers, *n* = 296 children of imprisoned mothers, and *n* = 1032 children with an imprisoned father or mother; and excluding from analyses of violent paternal, maternal and biparental offending, respectively: *n* = 1474 children of imprisoned fathers, *n* = 210 children of imprisoned mothers, and *n* = 380 children with an imprisoned father or mother). Results were unchanged; full details are available from the authors on request.

5 To identify any associations for which girls and boys differed significantly in the effect of parental offending on developmental outcomes, further analyses in the total sample were completed with the inclusion of a sex × offending interaction term. Few significant interactions were observed, and almost exclusively for the PHYSICAL domain, where for all six offending exposures, girls were characterized by significantly greater magnitudes of effect than boys. The association of any maternal offending with the EMOTIONAL domain was also significantly greater in girls than boys.

6 Among children with a history of any paternal offending, one-fifth (20.4%) also had a mother with a history of any offending, whereas almost two-thirds (62.3%) of children with a history of any maternal offending also had a father with similar offending history. For violent offending, the corresponding rates were 12.7 and 50.5%, respectively.

7 The percentage of children who presented vulnerability on at least two of the specific AEDC domains ranged between 1.7% for children with vulnerability on the EMOTIONAL and COGNITIVE domains (such that 23.2% of children with COGNITIVE vulnerability also had EMOTIONAL vulnerability) up to 4.4% of children with vulnerability on the SOCIAL and EMOTIONAL domains (such that 61.4% of children with SOCIAL vulnerability also had EMOTIONAL vulnerability).

## Footnotes

† The notes appear after the main text.

## Supplementary material

The supplementary material for this article can be found at https://doi.org/10.1017/S0033291716003007
